# Supplementary material for: Preparation of Active Peptides from Camellia vietnamensis and Their Metabolic Effects in Alcohol-Induced Liver Injury Cells
Source: Molecules. 2022 Mar 9;27(6):1790. doi: 10.3390/molecules27061790 (PMC8951368; doi:10.3390/molecules27061790)
Supplement: Supplementary file 1 [file molecules-27-01790-s001.zip › molecules-1591241-supplementary.pdf]

Supplementary Material

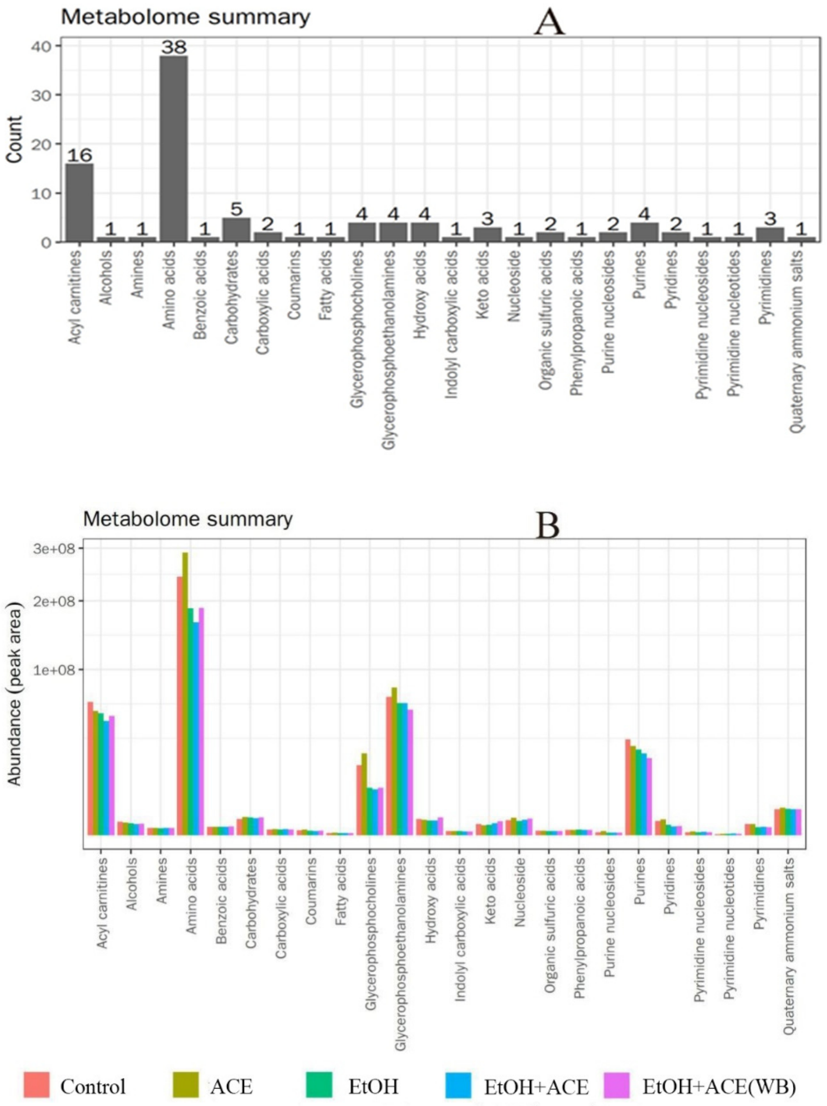

Figure S1: Statistics chart of L-02 cell metabolites(**A**:Metabolites in L-02 cells; **B**:Average content of each type of metabolites in each group)
